# Dual monoclonal antibody-based sandwich ELISA for detection of in vitro packaged Ebola virus

**DOI:** 10.1186/s13000-018-0773-1

**Published:** 2018-12-19

**Authors:** Junjie Zai, Kai Yi, Lilan Xie, Jiping Zhu, Xiaoting Feng, Yaoming Li

**Affiliations:** 1 Hubei Engineering Research Center of Viral Vector, Applied Biotechnology Research Center, Wuhan University of Bioengineering, Wuhan, 430400 China; 2 College of Life Science and Technology, Wuhan University of Bioengineering, Wuhan, 430400 China

**Keywords:** Monoclonal antibody, Ebola virus, GP, Detection, ELISA

## Abstract

**Background:**

Rapid transmission and high mortality of Ebola virus disease (EVD) highlight a urgent need of large scale, convenient and effective measure for Ebola virus screening. Application of monoclonal antibodies (mAbs) are crucial for establishment of an enzyme-linked immunosorbent assay (ELISA) with high sensitivity and specificity.

**Methods:**

The traditional cell fusion technique was used to generate a panel of hybridomas. Two mAbs were characterized by SDS-PAGE, Western blot, Indirect immunofluorescence assay (IFA). A sandwich ELISA was established using the two mAbs. The detection capability of the ELISA was evaluated.

**Results:**

In the current study, we produced two murine-derived mAbs (designated as 6E3 and 3F21) towards Zaire Ebola virus glycoprotein (GP), the major viral transmembrane spike protein associated with viral attachment. It was shown that 6E3 and 3F21 recognized GP1 and GP2 subunits of the GP respectively. Furthermore, 6E3 and 3F21 bound to corresponding epitopes on GP without reciprocal topographical interpretation. Subsequently, a sandwich ELISA based on the two mAbs were established and evaluated. The detection limit was 3.6 ng/ml, with a linear range of 3.6–100 ng/ml. More importantly, Ebola virus like particles (eVLPs) were able to be detected by this established virus detection measure.

**Conclusions:**

We produced and characterized two murine-derived mAbs (designated as 6E3 and 3F21) towards Zaire Ebola virus glycoprotein (GP), and established a sandwich ELISA based on the mAbs. It was suggested that the sandwich ELISA provided an alternative method for specific and sensitive detection of Ebola virus in the field setting.

## Background

Ebola virus infection results in severe hemorrhagic fever in humans with high case-fatality rates of up to 90% [1]. Over 11,000 deadly cases occurred during the 2013–2018 West African Ebola virus disease (EVD) epidemic. EVD causes a significant public health threat for affected region and for previously unaffected areas, as manifested by the increase in the incidence of EVD outbreaks over the past twenty-five years [2]. Therefore, the deathly epidemic emphasized a emergence for rapid, sensitive, reliable diagnostic method to monitor and control the early viral spread.

There were five distinct species of EBOV: Zaire ebolavirus (ZEBOV), Sudan Ebola virus (SEBOV), Ivory Coast Ebola virus (ICEBOV), Reston Ebola virus (REBOV), and Bundibugyo Ebola virus [3]. Ebola virus is enveloped, non-segmented, negative-stranded RNA virus belonging to the family Filoviridae, which consists of seven structural proteins: nucleoprotein (NP), viral protein 35 (VP35), VP40, glycoprotein (GP), VP30, VP24, and polymerase [4, 5]. These viral components are essential for progeny virus replication and assembly, and are also becoming the targets for viral infection monitoring [6, 7]. For the very early diagnosis of EVD in suspected cases, detection of viral genome RNA by quantitative real-time RT-PCR (qRT-PCR) is recommended [7–9], which however requires laboratory operations, skilled technicians and special equipments/facilities. Currently, there are three rapid Immunochromatography (IC) diagnosis assays for Ebola virus screening approved by the FDA and/or the WHO: ReEBOV (Corgenix), OraQuick Ebola (OraSure Technologies), and SD Q Line Ebola Zaire Ag (SD Biosensor) [10]. IC assays using filovirus-specific monoclonal antibody (mAb) is rapid and simple antigen-detection test for early diagnosis of viral infections in the field setting, however they are unable to detect samples in large scale, and in general with lower sensitivity than enzyme-linked immunosorbent assay (ELISA).

Accumulating studies revealed that two virus-specific mAbs were used to set up an effective ELISA kit for pathogen diagnosis [11, 12]. In this study, we intensively described the properties of two selected mAbs against Ebola virus GP spike protein and set up a sandwich ELISA based on the two mAbs, followed by evaluating its detection capability. It was suggested that the sandwich ELISA could be used as a promising measure for Ebola virus detection.

## Methods

### Cell, immunogen, and plasmids

HEK 293 T, Vero 1008 and RD cells were cultured in Dulbecco’s modified Eagle’s medium (Invitrogen, Carlsbad, CA), supplemented with 10% heat-inactivated fetal bovine serum (FBS) (HyClone, Logan, UT), and penicillin/streptomycin at 37 °C in a 5% CO_2_ humidified atmosphere. Recombinant Zaire Ebola virus GPdTM (0501–015, IBT BIOSERVICES,USA) was mature, recombinant, His-tagged Zaire Ebola virus Glycoprotein minus the transmembrane domain (rZEBOV GPdTM). Plasmid pEAK13-GP (Zaire) expressing Zaire Ebola virus GP was a gift from Dr. C Jiang, Tsinghua University, China [13]. Plasmids expressing VP40, GP1, or GP2 (Zaire) were constructed in our laboratory. MAb 2G4,which was specific to Ebola virus GP2, was kindly provided by George F. Gao, Chinese Center for Disease Control and Prevention, China.

### Preparation of monoclonal antibodies against Ebola virus GP

The preparation of Ebola virus GP-specific mAbs were generated as previously described [14]. In brief, 5-week-old female BALB/c mice 6–8 weeks old were immunized 50 μg rZEBOV GPdTM with 3-weeks’ interval. At four weeks after the last booster and 3 days before cell fusion, the mice were boosted with 200 μg of the rZEBOV GPdTM. Three days later, mice splenocytes were harvested and fused with SP2/0 using 50% polyethyleneglycol (Sigma-Aldrich, MO). Hybridoma was screened using indirect ELISA. The positive hybridoma cells were cloned by a limiting dilution and the stable hybridoma clones were injected into liquid paraffin-pretreated abdominal cavities of BALB/c mice. Subsequently, the mAbs were harvested and purified from the seroperitoneum with an antibody purification kit according to the manufacturer’s specifications (NAb™ Protein A/G Spin Kit, Thermo Scientific, USA).

### Western blot

Western blot analysis was performed as described previously with minor modification [15]. HEK 293 T cells were seeded in 35-mm glass-bottom dishes and transfected with the plasmid expressing GP1 or GP2. At 24 h posttransfection, tansfected cells were subject to 12% SDS-PAGE and transferred to PVDF membranes (0.45 mm, Millipore) followed by blocking with 5% nonfat milk in PBST and probed with appropriate primary mAbs at room temperature (RT) for 2 h. After washing three times with PBST, the membrane was incubated with horseradish peroxidase-conjugated goat anti-mouse IgG (1:8000, SouthernBiotech, USA). The results were developed using an enhanced chemiluminescence Western blot detection system (Amersham, Little Chalfont, UK) and exposed to X-ray film.

### Dot-blot

Dot-blot analysis was performed as described previously [16]. Different amounts of protein (GP or HIV-1 GP120) (100, 10, 1, 0.1, and 0.01 ng) were dropped onto a nitrocellulose (NC) membrane, then the membrane was incubated for 2 h at RT. The NC membrane hybridized with mAbs (1 μg/ml) for 1 h at RT. After 4 times’ washes in PBST, the membrane was incubated for 30 min at RT with horseradish peroxidase-conjugated goat anti-mouse IgG (1:8000, Southerbiotech, USA) in PBS. Then, after 6 times’ washes, the blots were developed using an enhanced chemiluminescence Western blot detection system (Amersham, Little Chalfont, UK) and exposed to X-ray film.

### Biolayer Interferomtery

Binding assays were performed in 96-well microplates by Octet Red system (Ferbio) [17]. Firstly, APS sensors were rinsed in PBS. Secondly, APS sensors were coupled with 200 μl PBS with mAbs (1 μg/ml). Thirdly, APS sensors were moved into PBS buffer and incubated to clear unabsorbed mAbs. Lastly, APS sensors were exposed to GP at concentrations of 1 μg/ml. Association was monitored for 1800 s followed by dissociation in PBS alone for another 1800 s. The standard curve was measured at the beginning and the end of the assay to confirm that it was reproducible and valid over the time taken to run all rows of samples. Data were processed automatically using the Octet User Software version 3.1.

### ELISA additive tests

The additive test analysis was performed as described previously [18]. AI = {[2 × A1 + 2/(A1 + A2)] − 1} × 100, where A1 and A2 were the ODs obtained when the mAbs were assayed separately, and A1 + 2 was the OD when the same amounts of the two mAbs were pooled in the same well. Provided the concentrations of the mAbs were saturated for the GP protein, the Al would be negligible if both mAbs were detected at the same epitope and close to 100 when the two epitopes were topographically unrelated. The lowest AI reported for mAbs at different epitopes on GP was considered as the threshold for evaluating epitopic correlation.

### Indirect immunofluorescence assay (IFA)

IFA was performed as described previously [19]. HEK 293 T cells were seeded in 35-mm glass-bottom dishes and transfected with the plasmids expressing GP and VP40. At 24 h posttransfection, cells were fixed with 4% paraformaldehyde, and permeabilized with 0.2% Triton X-100. After three washes with PBS, cells were blocked in PBS containing 5% BSA at 4 °C overnight. Thereafter, cells were incubated with 3F21 or 6E3 at concentration 1 μg/ml at 37 °C for 1 h, respectively. After three washes with PBST, cells were then incubated with FITC-conjugated goat anti-murine IgG. Finally, cells were washed and subject to incubation with antifluorescence quenching reagent (Beyotime, CN) and observed under a fluorescence microscope (Olympus IX51).

### Generation of Ebola virus like particles (eVLPs)

Generation of eVLPs was performed as described previously [20]. Briefly, Ebola virus like particles (eVLPs) were generated by cotransfection of plasmids containing the Ebola virus glycoprotein (GP) and matrix protein VP40 into 293 T cells in 100 mm glass-bottom dishes. After 72 h incubation, the supernatant was clarified and then purified by 20% sucrose cushion, and then the purified samples were subject to electron microscope analysis.

### Neutralization assay

To generate EBOV pseudotypes, 4 × 10^6^ 293 T cells were co-transfected with 10 μg of a pNL4–3.Luc.R-E- [17] and 10 μg of a DNA plasmid encoding entire EBOV-GP (pEAK13-GP) [13]. The efficacy was evaluated by EBOV-specific neutralizing mAb 4G7 [20]. Sera were two-fold diluted in 50 μl, and mixed with 10 TCID50 pseudovirus in 50 μl. Then the mixture was added to the 96-well plate culturing for 1 h, followed by application 1 × 10^4^ 293 T cell each well. At 48 h post infection (hpi), cells were subject to determine the luciferase activity by by a Luciferase Assay System according to the manufacturer’s instructions (Promega) using Tuner Biosystems Modulus II.

### Sandwich ELISA

Sandwich ELISA was performed as described previously with minor modification [21]. Briefly, the purified 6E3 (5 μg/mL) in coating buffer (40 mmol/L Na_2_CO_3_, 60 mmol/L NaHCO_3_, pH 9.6) were adsorbed to the surface of 96-well flexible microplates (Greiner Bio-one, Frickenhausen, Germany) at 4 °C overnight. After discarding coating buffer, samples were incubated in the microplates for 1 h at 37 °C. After washing 5 times with PBST, the plates were incubated for 45 min at room temperature with HRP-conjugated 3F21 (1:5000). After washing with PBST seven times, immunoreactivity was visualized by means of a TMB substrate system (KPL, Gaithersburg, MD) and the optical density values (OD_630nm_) were measured using an ELISA plate reader (Thermo Labsystems, MA).

## Results

### Production of hybridoma and generation of mAbs

**Fig. 1:**
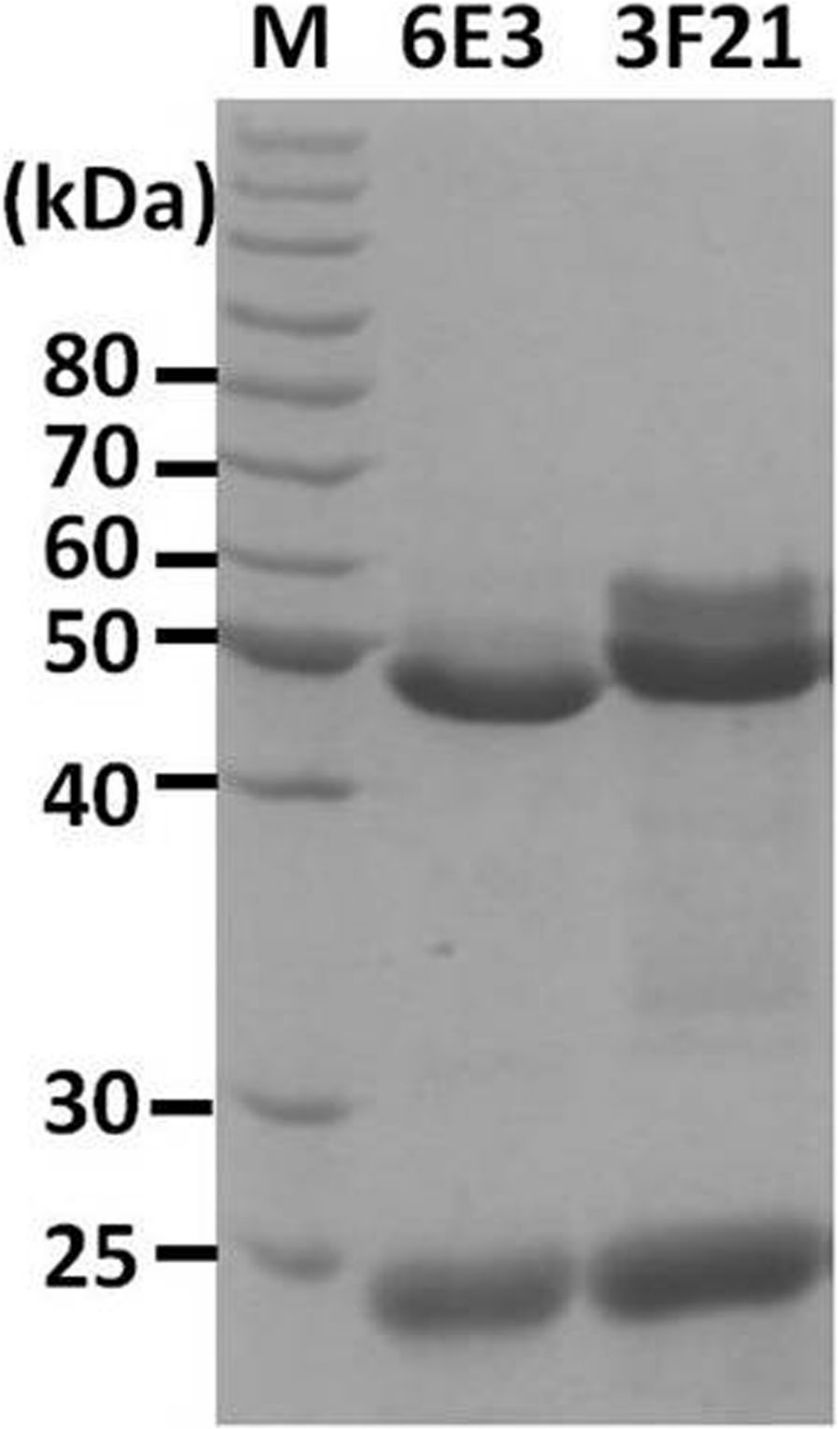
SDS-PAGE analysis of the purified mAbs. The mAbs of 6E3 and 3F21 were purified and analyzed as described. The two bands with a molecular weight of ~ 25 kDa and ~ 50 kDa respectively in each lane correspond to the light- and heavy-chain of that mAb

In order to achieve mAbs towards Ebola virus GP, we used commercially available recombinant Zaire Ebola virus GPdTM (Glycoprotein minus the transmembrane domain) to immunize BALB/c mice. Subsequently murine-derived hybridoma cells were generated by traditional cell fusion technology [14, 22]. Consequently, a panel of hybridoma cells were obtained, and the properties of the selected two murine IgG mAbs (designated as 6E3 and 3F21) were intensively characterized as below. SDS-PAGE result showed that mAbs 6E3 and 3F21 were with typical heavy chains ~ 50 kDa and light chains about ~ 25 kDa in molecule mass (Fig. 1).

### Characterization of mAbs 6E3 and 3F21

**Fig. 2:**
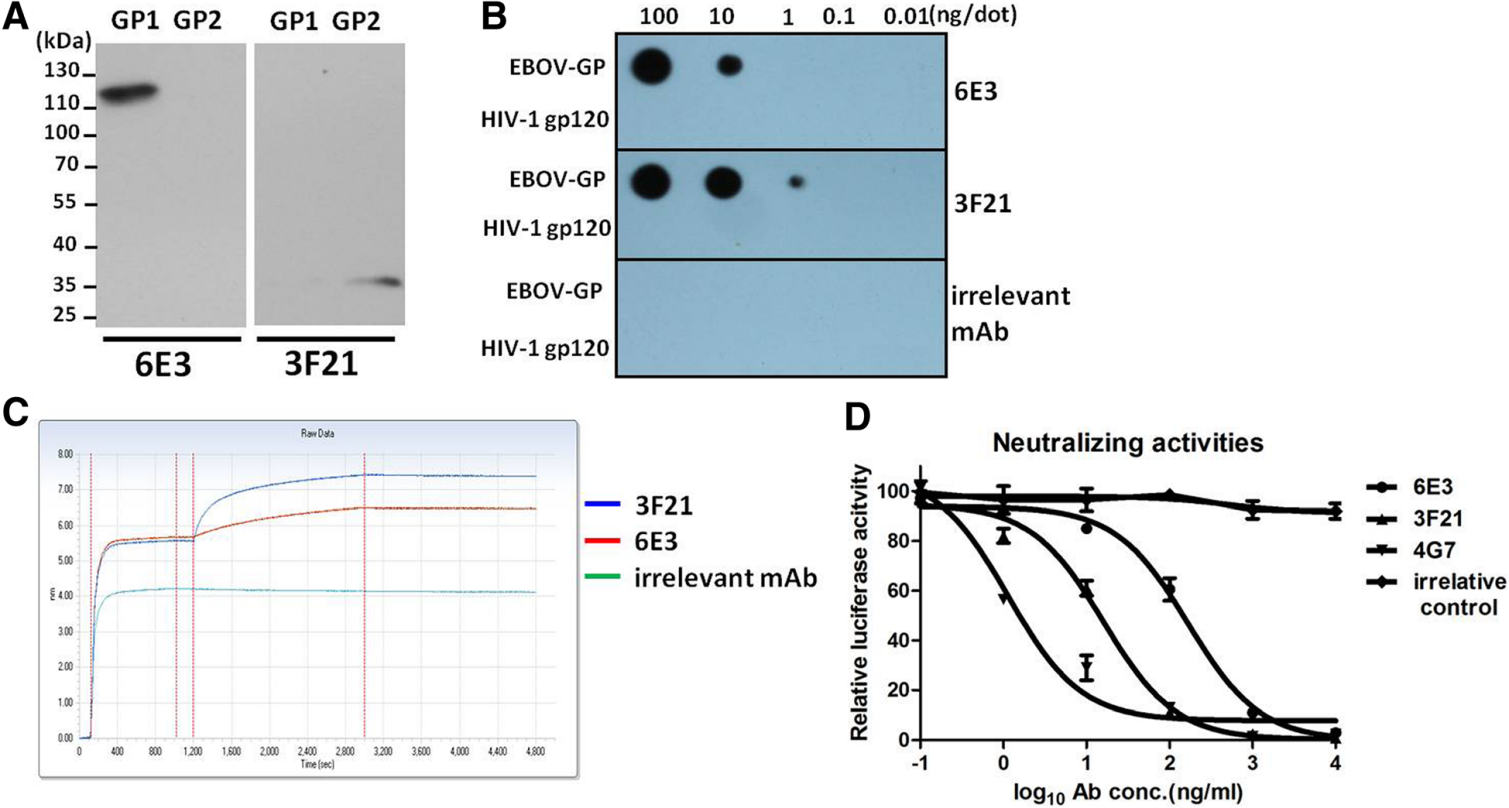
Characterization of the mAbs 6E3 and 3F21. **a**. Western blot showed that 6E3 and 3F21 recognized GP1 and GP2, respectively. **b**. Dot-blot results showed that 3F21 could recognize 1 ng GP protein, whereas 6E3 could only recognize 10 ng GP protein per dot. **c**. Molecular interaction analysis between GP and mAbs 3F21 and 6E3 showed that the affinity of 3F21 towards GP was stronger than that of 6E3 at the same antibody concentration. **d**. Neutralizing capacities of 6E3 and 3F21 were evaluated by EBOV pseudovirus. 4G7 served as a neutralizing mAb positive control. 5G10 acted as an irrelative antibody control

The subunits recognized by 6E3 and 3F21 were determined by Western blot. HEK 293 T cells were transfected with plasmids expressing GP1 or GP2 for 48 h. Cell lysates from transfected cell were analyzed by Western blot using mAb 6E3 or 3F21. As shown in Fig. 2a, mAbs 6E3 and 3F21 appeared to recognize GP1 (~ 120 kDa) and GP2 (~ 35 kDa), respectively.

The antibody affinity towards GP was primarily assessed by Dot-blot. rGPdTM, as well as HIV-1 GP120, were dropped onto the NC membrane for 2 h, mAbs 3F21, 6E3 and irrelative 5G10 were used as detector antibody. The results showed that 3F21 and 6E3 were able to specifically bind to Ebola virus GP (Fig. 2b). Moreover, mAb 3F21, but not 6E3, could detect 1 ng of GP per dot, indicating a better sensibility of 3F21 than that of 6E3 (Fig. 2b).

A binding assay based on Biolayer Interferometry was performed to further study the interaction between the mAbs and GP. rGPdTM was loaded to APS biosensor and the sensor tip was transferred to mAbs (6E3 and 3F21) or irrelevant antibody 5G10. The layer thickness (in nm) on APS sensor reflected the interaction between molecules. It was observed that a rapid and direct interaction occurred between mAbs (6E3 or 3F21) and GP. Moreover, the affinity of 3F21 towards GP was stronger than that of 6E3 at the same antibody concentration (Fig. 2c), which was in agreement with the result in Fig. 2c.

An EBOV pseudovirus system was used to evaluated the neutralizing capacities of 6E3 and 3F21. MAbs were 10-fold diluted and incubated with pseudovirus followed by assessment of luciferase activities compared with negative control. As shown in Fig. 2d, both 6E3 and 3F21 could suppress EBOV attachment to 293 T at relatively higher concentration compared with 4G7, which indicated two mAbs (6E3 and 3F21) could bind to native EBOV. MAb 4G7 served as positive control here [20].

### Epitope determination for 6E3 or 3F21

**Fig. 3:**
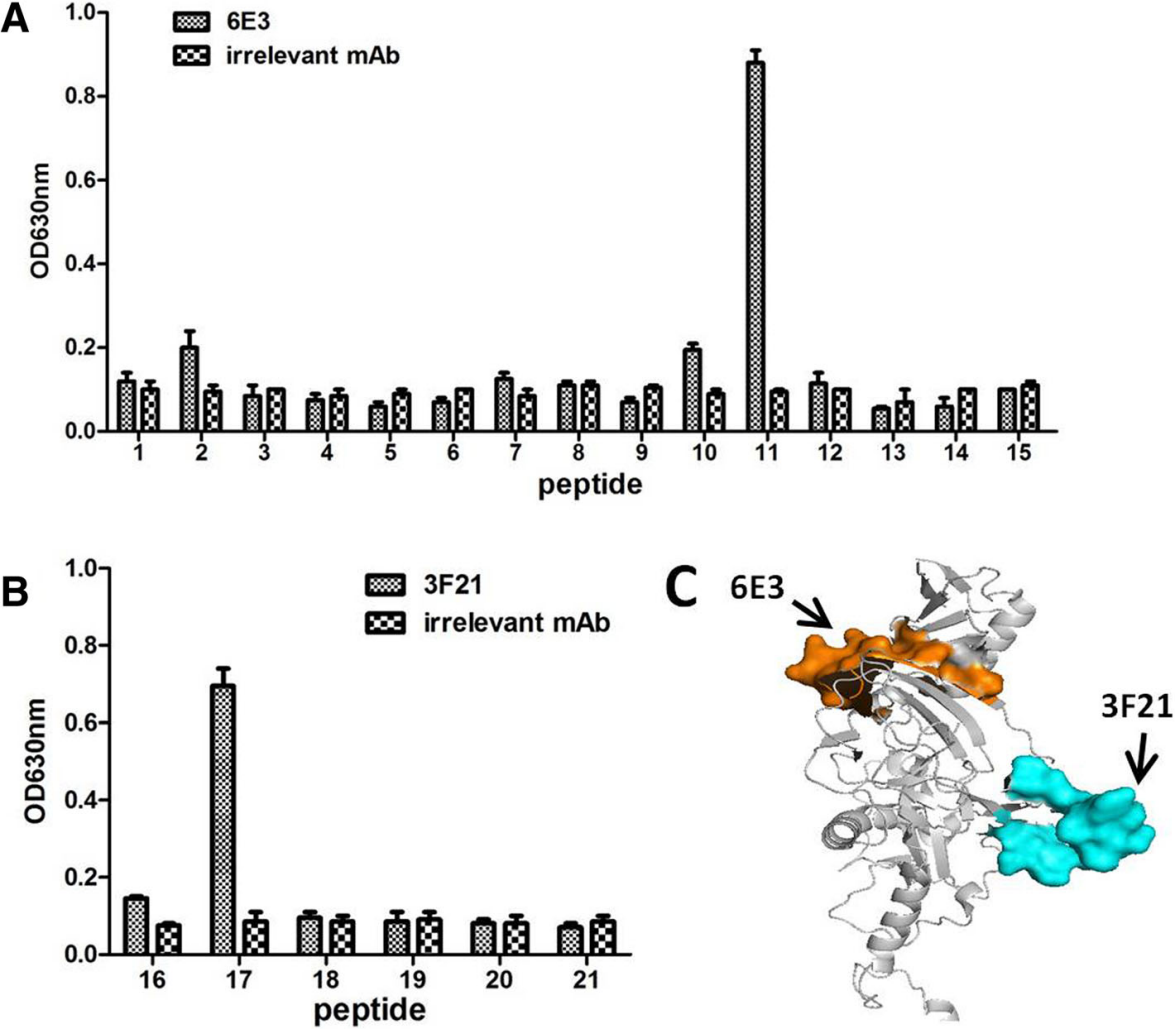
Epitope identification. **a**. Synthesized overlapping peptides were used to identify the accurate epitopes of 6E3 and 3F21. 6E3 recognized peptide-11 that was TIRYQATGFGTNEAEYL, whereas 3F21 recognized peptide-11 that was TQDEGAAIGLAWIPYFGPAA (**b**). The putative positions targeted by 6E3 and 3F21 were highlighted in orange and in cyan

Multiple synthesized overlapping peptides were used to determine the accurate domains on GP towards 3F21 and 6E3 by direct ELISA. ELISA microplate was coated with peptide at concentration of 5 μg/ml at 4 °C overnight. The 3F21, 6E3 and irrelevant 5G10 were used as detector antibody. The results showed that 6E3 recognized the peptide-11 (TIRYQATGFGTNEAEYL) (Fig. 3a), whereas 3F21 recognized peptide-17 (TQDEGAAIGLAWIPYFGPAA) (Fig. 3b). Moreover, we located the putative domains recognized by 6E3 and 3F21 on a GP structure model (PDB: 5JQ3) using PyMOL. It was observed that two epitopes of 6E3 and 3F21 were both exposed on the surface of the intact GP and widely separated (Fig. 3c).

An additive index (AI) test was performed to identify whether there was reciprocal spatial interpretation between the two antibodies 3F21 and 6E3 when binding to GP at the same time. ELISA plate was coated with GPdTM at concentration of 0.5 μg/ml (lower concentration) at 4 °C overnight. The 6E3 and 3F21 were used as detector antibody. Additive index value (97) between 6E3 and 3F21 was close to 100 (Table 1). The result showed that no antibody interpretation existed between 6E3 and 3F21 when binding to the GP, which was consistent with the results in Fig. 3c.

**Table 1:** Analysis of epitopes defined by mAbs against GP protein of Ebola virus

mAb	6E3	3F21
6E3	4^a^ (7^b^)	97 (2)
3F21		7 (2)

^a^Additivity Index

^b^Standard deviation

### 3F21 and 6E3 recognized in vitro packaged Ebola virus like particles (eVLPs)

**Fig. 4:**
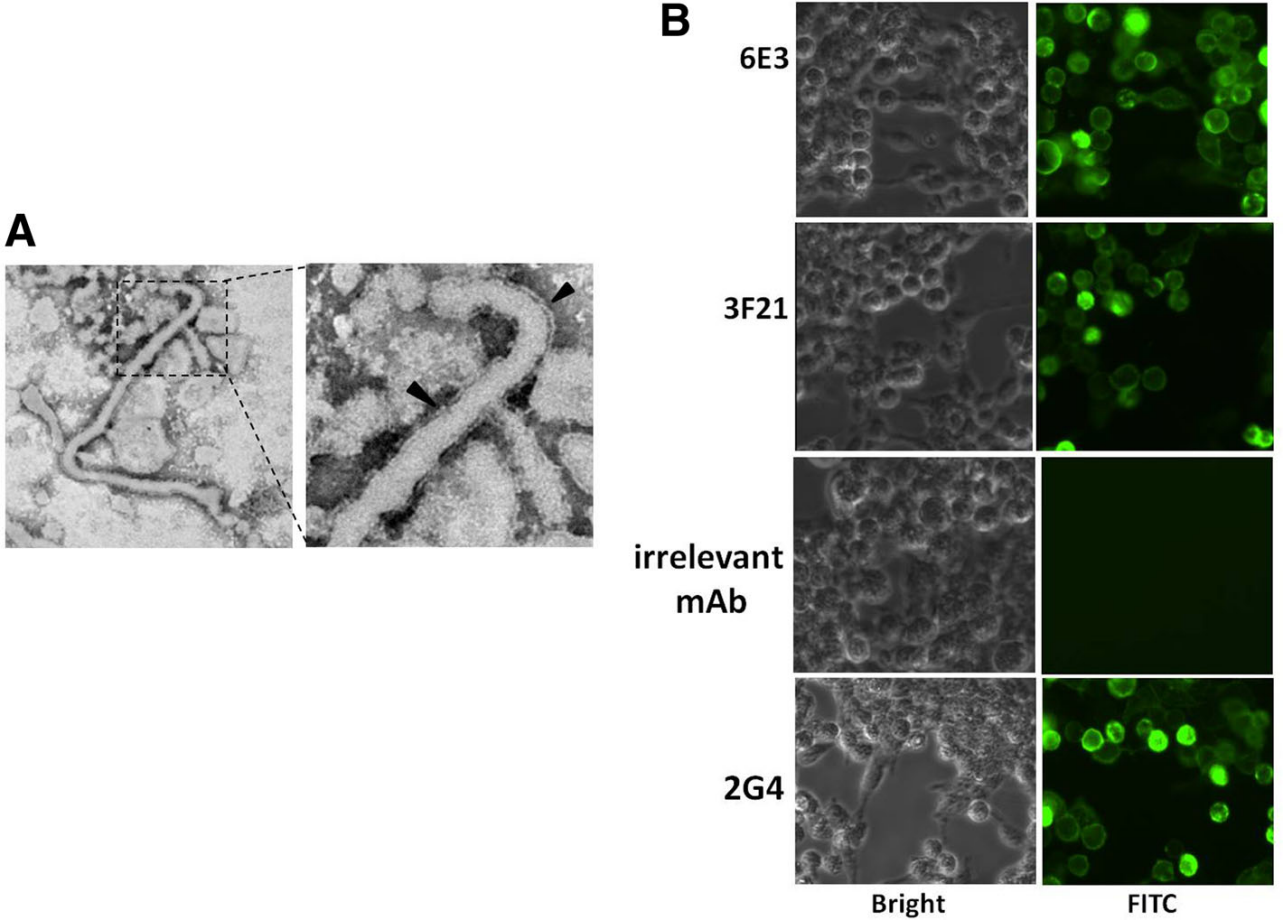
eVLPs were assessed by IFA. pEAK13-GP and pCD-VP40 were cotransfected to HEK-293 T, at 48 h of transfection, the supernatants were subject to electron microscope scan. **a**. The typical filavirus formation was observed. The arrowheads indicated virus membrane. **b**. The IFA results indicated that mAbs 3F21 and 6E3, like positive control 2G4, bound to eVLPs

HEK 293 T cells were cotransfected with plasmids expressing GP and VP40 for 72 h. The supernatant was clarified and purified by 20% sucrose cushion. The purified samples were subject to electron microscope analysis. The result showed that typical filavirus formation were successfully observed (Fig. 4a).

In IFA, HEK 293 T cells were cotransfected with plasmids expressing GP and VP40 for 48 h, followed by fixation and permeation. MAbs 6E3, 3F21, 2G4 and irrelative antibody 5G10 were used as detector antibody. 2G4 here served as a positive control [20]. The results showed that mAbs 3F21 and 6E3, like 2G4, were able to bind to eVLPs (Fig. 4b).

### The pair of mAbs was able to be used to develop an Ebola virus detection kit

**Fig. 5:**
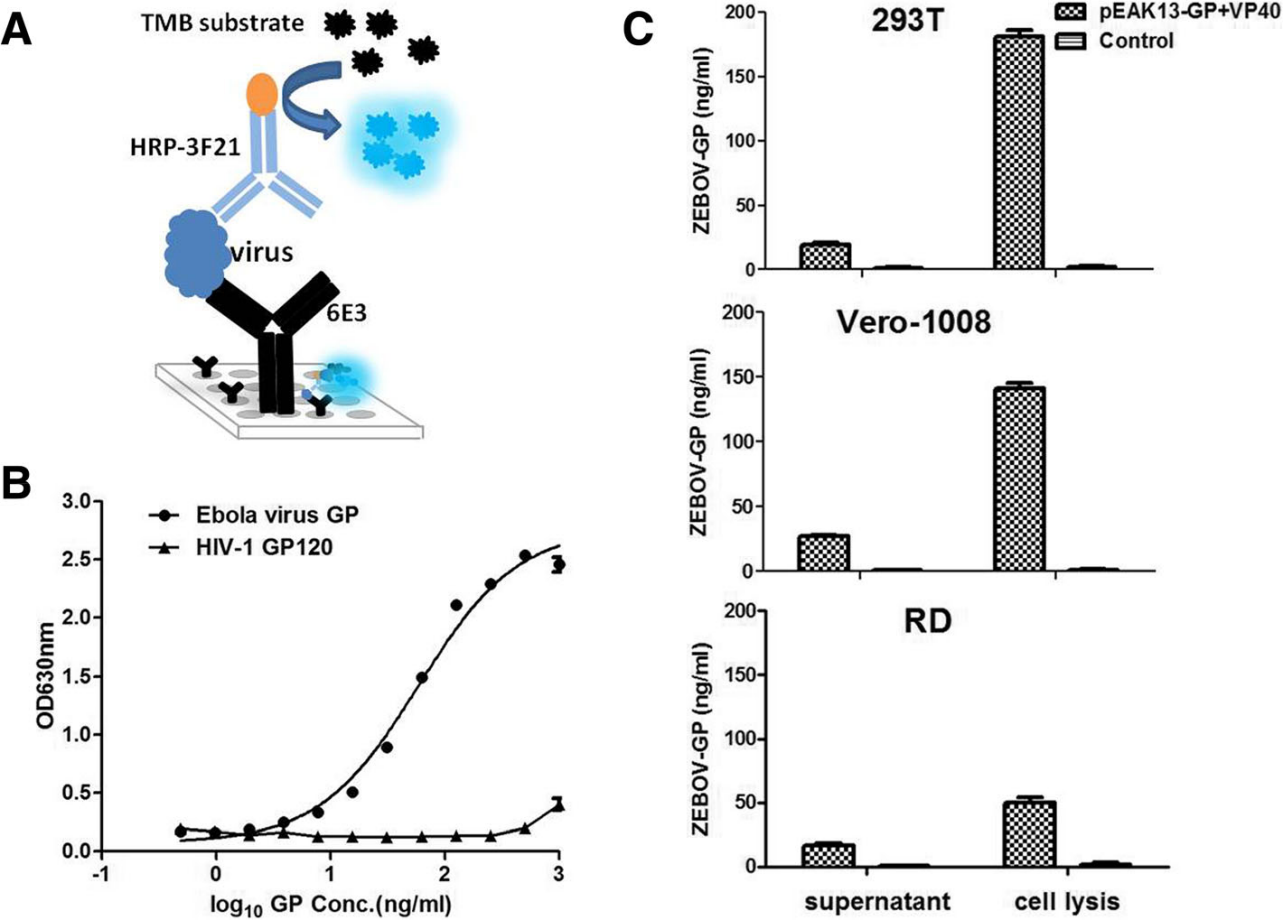
ELISA was used to detect in vitro packaged eVLPs. **a** Schematic sandwich ELISA for GP detection using dual mAbs. **b**. Establishment of sandwich ELISA using GP as reference standard. **c**. Quantitative determination of eVLPs in different cells (HEK 293 T, Vero 1008 and RD), which were cotransfected with plasmids expressing GP and VP40

Given that 3F21 and 6E3 were able to associated with eVLPs and were topographically unrelated, a sandwich ELISA was primarily designed based on the two mAbs for virus detection, in which 6E3 was used as capture antibody, whereas HRP-3F21 was used as detector antibody (Fig. 5a). The commercial rGPdTM were utilized as reference standard during establishment of sandwich ELISA. The result showed minimum quantity of rGPdTM about 3.6 ng/ml could be detected with the ELISA, with a linear range of 3.6–100 ng/ml (Fig. 5b).

HEK 293 T, Vero 1008, or RD cells were cotransfected with plasmids expressing GP and VP40 for 48 h, respectively. Supernatants were subject to quantitative determination of eVLPs with the ELISA. The result showed that ~ 25 ng/ml of GP appeared in the transfected cell supernatant, whereas there were ~ 200 ng/ml of GP observed in the transfected cells (Fig. 5c). In summary, 3F21 and 6E3 could be used to set up a sandwich ELISA for Ebola virus detection.

## Discussion

Effective and rapid detection method is crucial for the pathogen screening and for restriction of the virus rapid spread. In the current study, we obtained and characterized two murine-derived mAbs specifically towards Ebola virus GP, and established a sandwich ELISA based on the antibodies, which are able to detect Ebola virus like VLPs (eVLPs).

The Ebola virus glycoprotein (GP), which interacts with host Niemann-Pick C1 (NPC1) receptor, mediates viral attachment and entry into host cells and is the major inducer for the host immune response [23]. During Ebola virus replication, GP is proteolytically cleaved into GP1 and GP2 by host furin protease, which subsequently are disulfide-linked to form a mature spike GP. Several vaccine candidates are in development, most of which use the GP as the immunogen [1, 24]. Meanwhile GP as the most important component of virus was also the important target in Ebola virus detection and in producing neutralizing antibody [25]. Qiu et al., used recombinant VZV-Ebola GP, followed by GP protein to immune mouse, achieved several cell lines against Zaire Ebola virus, targeting different subunits (GP1 or GP2), without further analysis about antibody properties [20]. Han et al., utilized pCAGGS-GP and GP protein to immune mouse and obtain several mice, however they did not provide detailed information of the GP-specific mAbs [26]. Here we applied the recombinant GP to immunize mice and achieved several mAbs, furthermore we identified the mAbs in multiple aspects, including binding activities, affinities, accurate epitopes. More importantly, we found that the in vitro packaged eVLPs was able to be successfully detected, which indicated that the mAbs could recognize native Ebola virus particle.

Aim to establish a mAb-based antigen detection method with sandwich ELISA, the selected mAbs should be topographically unrelated. Thus the ELISA additive index test was used to determine whether or not the epitopes recognized by the different mAbs were overlapping [18]. The Fig. 3 results demonstrated that the epitopes detected by the mAbs (3F21 and 6E3) were reciprocally separated. Strikingly, the AI value further confirmed the notion that mAbs display no reciprocal topographical interpretation when complete binding to a GP molecule (Table 1). Therefore the two antibodies were able to be used to assemble a sandwich ELISA kit as capture antibody and detector antibody, respectively.

Dual mAs were helpful to enhance the specificity of the virus detection and monitoring. ReEBOV Antigen Rapid Detection Test (RDT) was the first RDT listed by the WHO. Following laboratory analytical validation as per the Food and Drug Administration guidelines, the specificity of this VP40-detecting test was evaluated at 95% on serum specimens and at 97% on whole blood specimens [27]. Its LDT was 4.8 ng/ml, which was essentially equal to our result (3.6 ng/ml). In that approved ReEBOV RDT, polyclonal antibody and mAb were involved in the kit as capture antibody and detector antibody. It was deduced that the application of mAb, instead of polyclonal antibody, likely enhanced the specificity of the assay.

Due to biosafety concerns, we were not allow to manipulate infectious Ebola virus. We hence had to evaluate the detection capacity of the ELISA by testing eVLPs, instead of active Ebola virus. Despite successful observation (Fig. 4) and detection (Fig. 5) of intact eVLPs, native infectious virus should be further estimated using this ELISA in the future.

## Conclusions

In summary, two murine-derived mAbs towards distinct antigenic sites of Zaire Ebola virus GP was developed. Based on the two mAbs with unique properties a sandwich ELISA was established for Ebola virus detection. We would take this research further by developing a more sophisticated system using these mAbs, aiming for a more accurate and convenient detection.

## Abbreviations

eVLPs: Ebola virus like particles
HRP: Horseradish peroxidase
mAb: Monoclonal antibody
